# To Detect Adulteration of Milk

**Published:** 1890-10

**Authors:** 


### TO DETECT ADULTERATION OF MILK.

The milkman who waters his goods generally does so under the impression that the water poured in incorporates itself with the milk, and cannot be detected except upon chemical analysis. This shows gross ignorance. The milk will hold only its own fluid; all foreign fluid will be precipitated if the mixture is allowed to stand for a couple of days. Any housewife may spot a dishonest milkman with very little trouble. Let her take a long, slender bottle, cleanse it thoroughly and let it dry out. If then it is filled with milk and allowed to stand in a cool, not cold, place for forty-eight hours, all the foreign fluid will be precipitated, that is, it will settle to the bottom of the bottle. The soured milk will then fill the middle of the bottle, and the fatty substance will be floating on top. Sometimes the top will be a layer of cream, then will come a layer of albumen, another artificial device to make the milk look rich ; then will come the soured milk, and at the bottom will be the foreign water The whole scheme of deception can be read by a glance at the bottle after one has had a single lesson in the rudiments of milk inspection. This sort of work is not scientifically satisfactory, but it will always develop the fundamental fact—whether or not the milk is normal.

